# Structurally-driven Enhancement of Thermoelectric Properties within Poly(3,4-ethylenedioxythiophene) thin Films

**DOI:** 10.1038/srep30501

**Published:** 2016-07-29

**Authors:** Ioannis Petsagkourakis, Eleni Pavlopoulou, Giuseppe Portale, Bryan A. Kuropatwa, Stefan Dilhaire, Guillaume Fleury, Georges Hadziioannou

**Affiliations:** 1 Laboratoire de Chimie des Polymères Organiques, CNRS - ENSCPB - Université de Bordeaux - UMR 5629, F-33607 Pessac, France; 2 Bordeaux INP, LCPO, UMR 5629, F-33600, Pessac, France; 3 Macromolecular Chemistry & New Polymeric Materials, Zernike Institute for Advanced Materials, Nijenborgh 4, 9747 AG Groningen, The Netherlands; 4 Laboratoire Ondes et Matière d’Aquitaine, CNRS - Université de Bordeaux - UMR 5798, F-33400 Talence, France

## Abstract

Due to the rising need for clean energy, thermoelectricity has raised as a potential alternative to reduce dependence on fossil fuels. Specifically, thermoelectric devices based on polymers could offer an efficient path for near-room temperature energy harvesters. Thus, control over thermoelectric properties of conducting polymers is crucial and, herein, the structural, electrical and thermoelectric properties of poly(3,4-ethylenedioxythiophene) (PEDOT) thin films doped with p-toluenesulfonate (Tos) molecules were investigated with regards to thin film processing. PEDOT:Tos thin films were prepared by *in-situ* polymerization of (3,4-ethylenedioxythiophene) monomers in presence of iron(III) p-toluenesulfonate with different co-solvents in order to tune the film structure. While the Seebeck coefficient remained constant, a large improvement in the electrical conductivity was observed for thin films processed with high boiling point additives. The increase of electrical conductivity was found to be solely in-plane mobility-driven. Probing the thin film structure by Grazing Incidence Wide Angle X-ray Scattering has shown that this behavior is dictated by the structural properties of the PEDOT:Tos films; specifically by the thin film crystallinity combined to the preferential edge-on orientation of the PEDOT crystallites. Consequentially enhancement of the power factor from 25 to 78.5 μW/mK^2^ has been readily obtained for PEDOT:Tos thin films following this methodology.

Thermoelectric (TE) materials have the potential to convert vast amounts of waste heat directly into electricity, therefore reducing the dependence on fossil fuel^1^. Combined with the rising need for clean energy, efficient thermoelectric materials are a subject of great interest^2^,^3^. In thermoelectrics, the concept of a phonon glass/electron crystal (PGEC) is often used to describe an ideal thermoelectric material. According to PGEC, a good TE material should inhibit the conduction of phonons (thus having a low thermal conductivity) while efficiently conducting electronic charge carriers. In order to quantify the efficiency of TE systems, the figure of merit, *ZT*, is used as a measure of performance. It is defined as *ZT* = *S^2^σT/κ*, where *S* is the Seebeck coefficient, *σ* is the electrical conductivity, *T* is the temperature and *κ* the thermal conductivity^1^. Materials engineering is offering unprecedented routes towards optimizing *ZT* by tuning the electronic and thermal properties, e.g. by introducing thermal boundaries and/or structural discontinuities at the nanoscale that effectively scatter phonons. Thus, values of *ZT* up to 2.2 have since been achieved with SnSe crystals over the last decade^4^. Alternatively, conducting polymers have recently gained momentum in the TE community for applications at room temperature^5^,^6^,^7^,^8^. Their great advantage is an intrinsically low thermal conductivity at room temperature (0.2–0.6 W/mK) that is complemented by their easy processability and their low cost. Thin films of poly(3,4-ethylenedioxythiophene) (PEDOT) derivatives doped with *p*-toluenesulfonate (Tos) molecules can exhibit a *ZT* as high as 0.25 at room temperature^6^, which is only 4 times lower than that of conventional Bi_2_Te_3_ at room temperature^2^,^3^,^4^, thus underlining the high potential of such systems for future applications.

For a given thermal conductivity, the figure of merit downscales to the power factor, *S^2^σ*, at a constant temperature leading to a valid method of materials comparison. The electrical conductivity can be further broken down for metals and doped semiconductors using *σ* = *eNμ*, with *N* being the carrier concentration, *μ* the carrier mobility and *e* the electron charge^9^. For the PEDOT:Tos system, most efforts to optimize the power factor have been focused on tuning *N*^6^,^10^. Note that *S* decreases with *N*, while *σ* increases at the same time^6^, rendering this optimization quite challenging. An alternative strategy is the enhancement of mobility while maintaining a constant *N*.

Transport properties of semiconducting polymers are driven by the polymer structure in the active layer^11^,^12^,^13^. For example, higher polymer crystallinity results in higher carrier mobility^14^. Several methodologies have been proposed in the literature for enhancing the degree of crystallinity for semiconducting polymers^15^. As far as the PEDOT-based systems are concerned, it was proved that the addition of dimethylsulfoxide (DMSO) in the dispersion delays the crystallization kinetics of PEDOT, thus resulting in an increased degree of crystallinity and a higher electrical conductivity^14^,^16^. Several other additives have been studied with respect to poly(3,4-ethylenedioxythiophene):poly(styrene sulfonate) (PEDOT:PSS), like ethylene glycol^5^ (EG) and dimethylformamide^17^ (DMF). For thermoelectric applications, PEDOT:Tos shows a higher potential since it exhibits a higher Seebeck coefficient with respect to PEDOT:PSS^7^. In this work we present, an thorough study of the effect of several solvent additives on the thermoelectric properties of PEDOT:Tos thin films. The additives were selected based on gradual increase of their boiling point temperatures, *T*_*b*_. Additives with a high boiling point are expected to reside in the film for longer time during film processing, acting as a plasticizer and promoting crystallization. In this way, we hope to increase hole mobility and, consequently, the electrical conductivity of PEDOT:Tos. A comprehensive characterization of these films has been performed, with respect to their thermoelectric, electrical and structural properties.

## Results & Discussion

**Figure 1:**
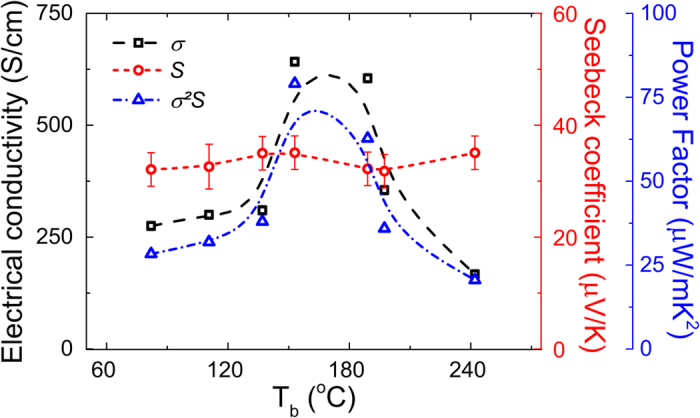
Thermopower data. S, σ, and the corresponding S^2^σ obtained for PEDOT:Tos thin films prepared with the various T_b_ additives.

In order to investigate the effect of solvent additives (see Table 1) on the thermoelectric properties, the Seebeck coefficient, *S*, was first measured and the results presented in Fig. 1. The processing of the PEDOT:Tos thin films with these additives was found not to significantly affect the resulting Seebeck coefficients, which has a constant value of 34 ± 5 μV/K. The electrical conductivity was also measured for the various films and, as apparent in Fig. 1, σ follows a bell-shaped curve with respect to the additive boiling point, exhibiting a maximum value of approximately 640 S/cm for DMF. Since *S* is constant, the power factor, *S^2^σ*, displays a similar bell-shaped trend as the electrical conductivity, with a maximum value at 78.5 μW/mK^2^ for DMF.

**Figure 2:**
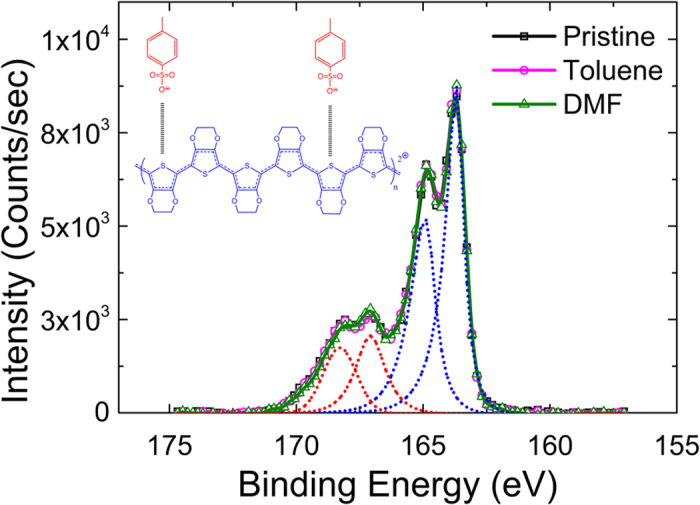
S(2p) XPS spectra of the reference PEDOT:Tos film (black curve) and those treated with toluene (magenta) and DMF (green). The red and blue dashed lines represent the fitted doublets for the Tos and EDOT units, respectively. Inset: chemical structure of PEDOT:Tos.

To understand the physical origin of this behavior, the dependence of *σ* with regards to *N* and *μ* has to be examined. In semiconducting polymers like the PEDOT-based ones, *N* is correlated to the oxidation level, since doping is achieved by oxidizing the polymer chain^6^,^18^. Oxidized sulfur atoms are subsequently stabilized by the anion of the tosylate counter-ion^19^. Holes act as the charge carrier of the system as proven by the positive Seebeck coefficients. Since *σ* can be tuned by either tuning *N* through doping and/or *μ*, probing the oxidation level of PEDOT:Tos thin films is an indirect way to investigate any modifications of the carrier concentration. X-ray photoelectron spectroscopy was used to determine the oxidation level of each sample. The PEDOT:Tos material has two different sulfur atoms; one in the tosylate counter-ion molecule (S_a_) and the other in the thiophene unit (S_b_). These two sulfur atoms have different chemical environments, therefore showing two distinct XPS signatures. S_a_ resides in a highly electronegative environment due to the presence of three surrounding oxygen atoms, yielding a signal measured at higher binding energies (168–170 eV), while the signal at low binding energies (163–166 eV) corresponds to the doublet of S_b_^6^,^20^. XPS spectra for sulfur S(2p) for three PEDOT:Tos distinct samples are presented in Fig. 2. For clarity we have chosen to present only the spectra acquired for the pristine PEDOT:Tos film (no additive used) and those treated with toluene and DMF; exhibiting conductivities of 230 S/cm, 300 S/cm and 640 S/cm, respectively. A perfect overlap of the three S(2p) spectra is observed, suggesting that there is no significant change in the oxidation level induced by treatment process. Quantification of the oxidation level was determined *via* the ratio of the S_b_ doublet area with respect to the S_a_ doublet area^6^,^20^. An oxidation level of 33% was calculated for all PEDOT:Tos thin films, which is in accordance with that previously reported in the literature for PEDOT:Tos^6^. It can therefore be concluded that the carrier concentration, *N*, of the system remains constant throughout. This implies that the measured changes in electrical conductivity are due to the mobility.

**Figure 3:**
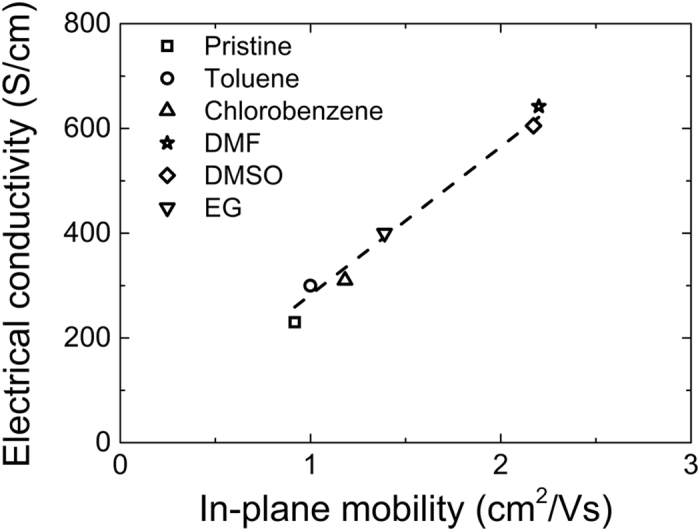
Plot of *σ* vs. *μ* for a series of PEDOT:Tos with additives. In accordance with the conductivity expression for doped semiconducting, *σ* is directly proportional to *μ*.

PEDOT-based polymers are heavily doped semiconductors due to their high carrier density^14^. Therefore, conventional transistors are not appropriate for the determination of the mobility of such polymers owing to the screening of the applied electric field by the charge carriers. For this reason, electrolyte gated transistors have been proposed in the literature as an alternative for the measurement of transport properties^14^,^21^,^22^. These transistors incorporate an ion gel as the dielectric layer, which modifies the density of charge carriers and allows the observation of field effects. Details on device fabrication, measurements of the transistor output characteristics and mobility calculation are provided in Figures S1 and S2. The results of the in-plane mobility of the selected films are summarized in Table 1. All calculated mobilities are comparable to the values reported in literature^14^,^21^. Logically, the in-plane mobility *versus* the additive boiling point plot (See Figure S3) presents a similar bell-shape as the one reported in Fig. 1 for conductivity. This underlines the direct influence of the mobility on the electrical conductivity behavior. To further assert the correlation between the measured *σ* and *μ*, the electrical conductivity versus the in-plane mobility was plotted in Fig. 3 for the systems under investigation. A linear dependence of the electrical conductivity with respect to hole mobility was observed, which is in accordance with the solid state equation *σ* = *eNμ*^9^. An estimation of the carrier concentration, *N*, was obtained from the slope of the linear fit. A value of 1.7 × 10^21^ carriers/cm^3^, in accordance with those reported in literature for PEDOT systems and heavily doped semiconductors (≈10^21^ carriers/cm^3^), was calculated^14^,^21^,^22^.

**Figure 4:**
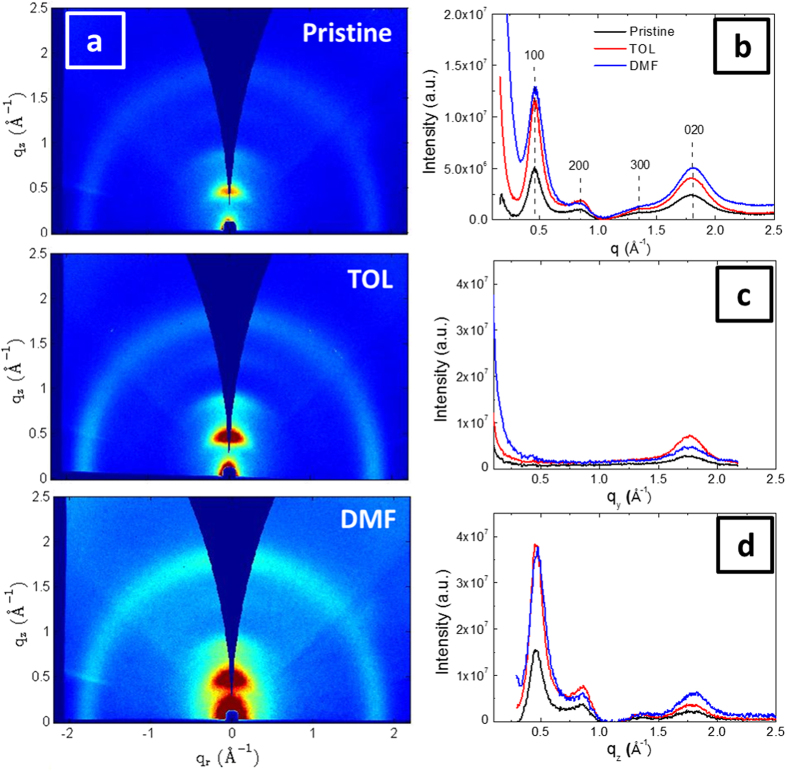
GIWAXS characterization of PEDOT:Tos thin films with additives. (**a**) 2D GIWAXS images recorded for the pristine PEDOT:Tos and those treated with toluene and DMF. The corresponding 1D scattering patterns: (**b**) the radially averaged intensity with respect to the scattering vector *q*, (**c**) the in-plane intensity line-cut and (**d**) the near out-of-plane intensity line-cut. In all cases the scattering intensity was normalized by film thickness and the substrate scattering was subtracted.

Transport properties of organic semiconductors are strongly related to their thin film morphology^11^,^12^,^13^,^15^,^16^,^23^,^24^,^25^,^26^. In order to understand the nature of the additive effect on mobility, the structural properties of the PEDOT:Tos thin films were probed by GIWAXS. The (*q*_*r*_, *q*_*z*_) 2D scattering pattern recorded for the pristine PEDOT:Tos film as well as those recorded for the toluene-treated and DMF-treated films are presented in Fig. 4a. We opt to present these data because there is a gradual increase in conductivity, from 230 S/cm for the pristine film, to 300 S/cm for toluene, to 640 S/cm for DMF. Clearly these films scatter anisotropically, with the majority of scattered intensity along the near out-of-plane *q*_*z*_ axis (with *q*_*r*_ ≈ 0), that is, perpendicular to the substrate. This is indicative of preferential orientation of PEDOT crystallites in the film which will be addressed in the next section. In Fig. 4b, the radially averaged scattered intensity is plotted with respect to the scattering vector *q*. Note that in all cases, the scattered intensity is presented after normalization for film thickness and background subtraction, to allow for quantitative comparison between samples. For all the PEDOT:Tos thin films, the strong peak at 0.44 Å^−1^ and the following at 0.88 Å^−1^ are attributed to the (100) and (200) reflections, in accordance to the assignment proposed by Aasmundtveit *et al*.^27^. The resultant repeat distance is 14.28 Å and corresponds to the unit cell length along the *a* axis that is the EDOT direction, perpendicular to the polymer backbone (aligned along the *c* axis). A schematic of the unit cell showing the directions of the *a, b* and *c* axes is provided in Figure S4. A third peak is observed at around 1.3 Å^−1^ that can be assigned to the (300) reflection. However the very low intensity of this peak suggests a limited long-range order along the *a*-axis, indicating a material with paracrystalline distortions. At 1.77 Å^−1^ a broad and intense peak is observed that is assigned to the (020) reflection and corresponds to the π-π stacking direction^27^.

**Figure 5:**
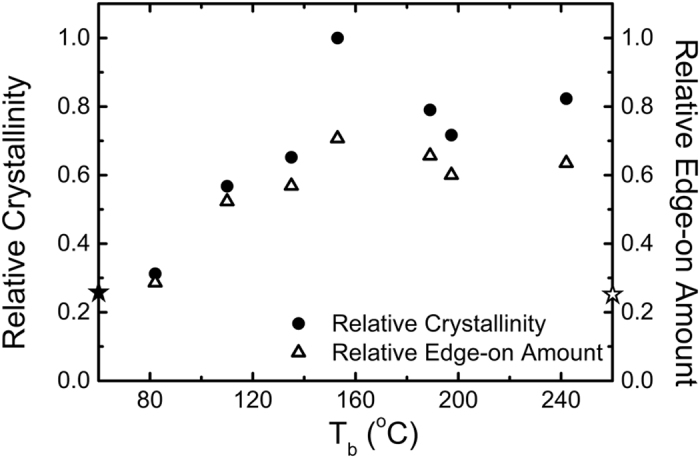
Relative crystallinity and relative amount of edge-on oriented crystallites as a function of the boiling point of the various additives. The star symbols on both axes correspond to the respective data obtained for the pristine PEDOT:Tos films. Crystallinity data were normalized by the value obtained for the most crystalline one.

Besides, an increase of the scattered intensity was observed when the PEDOT:Tos thin films were processed with the additives, particularly for the (100) and (020) peaks. Given the fact that the area below the scattering peaks is indicative of the degree of crystallinity - the higher crystallinity resulting in higher intensities - the pristine PEDOT:Tos film is less crystalline than those prepared with additives. Even if the area of the (100) peaks can lead to a measure of the relative crystallinity, a more rigorous analysis involves the *I(χ)* polar plots, *χ* being the polar angle defined with respect to the normal direction^28^. The area of the *I(χ)* × *sin(χ) vs χ* plots for the (100) peak (see Figure S5) was subsequently used as a measure of the relative crystallinity considering the PEDOT films as “in-plane powders” (i.e. isotropic in-plane orientation of the crystallites)^28^ and the results normalized with respect to the most crystalline sample are presented in Fig. 5.

The relative crystallinity increases with respect to the boiling temperature of the additive until a maximum for DMF followed by a lower plateau for the higher boiling point additives. This behavior supports our initial hypothesis that using additives with various boiling points can favorably alter the crystallization properties of the PEDOT:Tos films. Given that the main solvent of our dispersion (1-butanol) has T_b_ = 115 °C, introducing a higher boiling point additive, such as DMF or DMSO, would slow the evaporation rate of the solvents, delay the crystallization kinetics of the polymer chains and allow further crystallization. These observations are in agreement with other reports in the field of organic electronics^14^,^16^,^25^.

In order to examine the anisotropic nature of the 2D images, the in-plane and near out-of-plane line-cuts are presented in Fig. 4c,d (The whole set of 1D GIWAXS patterns recorded for the various additives is presented in Figure S6a–c). In the near out-of-plane pattern (*I*(*q*_z_) vs *q*_*z*_, Fig. 4d), we observe the (h00) group and the (020) reflection, similarly to the *I*(*q*) vs *q* pattern, while in the in-plane pattern (*I*(*q*_*y*_) vs *q*_*y*_, Fig. 4c) the (h00) reflections are absent and only the broad (020) reflection at 1.77 Å^−1^ is observed. The absence of the (h00) group from the in-plane pattern allows one to conclude that there is a preferential orientation of the crystallites in the film with respect to the substrate. It further indicates that the PEDOT chains are mainly oriented in an edge-on configuration, *i.e.* the thiophene rings of EDOT are preferentially aligned with their planes normal to the substrate. However crystallites oriented with face-on, *i.e.* with the thiophene ring parallel to the film plane, should still be present, since the (020) peak corresponding to π-π stacking is apparent in both line-cuts. Quantitative information on the crystallite orientation can be extracted from the *I(χ)* × *sin(χ)* vs *χ* plots (see Figure S5). In particular the fraction of edge-on oriented crystallites can be estimated from the ratio between the *I(χ)* × *sin(χ)* vs *χ* integral from 0° to 45° and the one from 0° to 90°. For all PEDOT:Tos thin films, the majority of the crystallites (between 0.75 and 0.9) are oriented edge-on which is consistent with the absence of the (100) peak in the in-plane line-cut (Fig. 4c).

**Figure 6:**
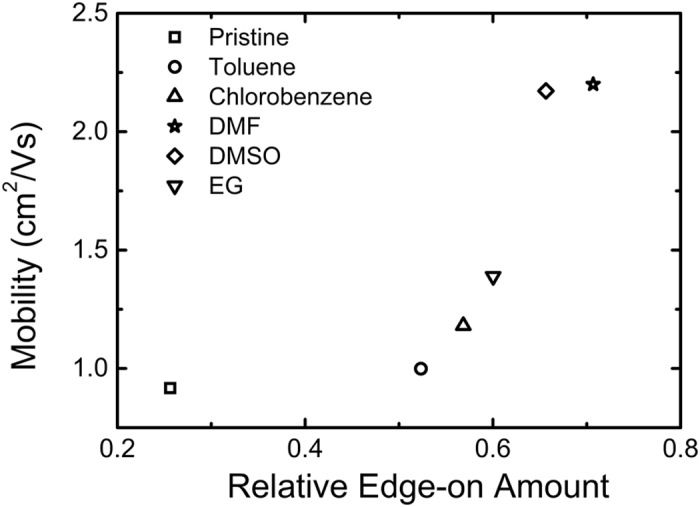
In-plane mobility of the PEDOT:Tos thin films with various high boiling point additives with respect to the amount of edge-on oriented crystallites.

For crystalline semiconducting polymers, both thin film crystallinity^7^,^12^,^14^,^15^ and chain/crystallite orientation^13^,^24^,^29^ influence the charge transport properties. In order to take into account both parameters, the product between the relative crystallinity with the edge-on fraction was calculated. This product has in fact a physical meaning, as it represents the amount of edge-on crystallites in the PEDOT:Tos thin films. Fig. 5 shows that the relative edge-on amount follows clearly the crystallinity progression. Since relative crystallinity has been taken into account, using this product as a structural parameter allows for direct comparison between different samples. Interestingly, a clear correlation exists between the mobility and the relative amount of edge-on crystallites, as shown in Fig. 6.

The increase in the in-plane mobility with the quantity of edge-on crystallites is in agreement with the current understanding of efficient charge transport in semiconducting polymers, where the main transport mechanism occurs by hopping between doped conjugated chains^13^. For conducting polymers like PEDOT, it is also accepted that charge transport is anisotropic, and is dictated by the presence of highly conducting crystalline “metallic” islands that are embedded in a less conducting amorphous polymer matrix^30^,^31^. Thus, systems with higher crystallinity are expected to exhibit higher mobility and therefore electrical conductivity. Furthermore, it has been shown by Sirringhaus *et al*.^13^ and Crossland *et al*.^24^ that charge mobility depends on the orientation of the crystalline lamellae of semiconducting polymers with respect to the measurement plane. In fact, reported mobilities of crystalline semiconducting polymers are three times higher along the polymer backbone (*c*-axis) with respect to that measured along the π-π stacking direction (*b*-axis), while that measured along the *a*-axis direction is a hundred times lower than that measured along the *b*-*c* plane. Therefore, in order to achieve high in-plane mobility, an edge-on orientation is necessary, where the chains are π-stacked along this plane and, thus, interchain and intrachain hopping transport along the backbone are promoted. Thus, a combination of the aforementioned structural parameters (i.e. edge-on orientation and crystallinity) is pivotal for polymer films with high carrier in-plane mobility. This is confirmed with the results displayed in Fig. 6; the films that contain the highest amounts of edge-on crystallites exhibit the highest mobility. To the best of our knowledge, this study is one of the very few reports regarding a quantitative correlation between transport properties and structural characteristics in polymer semiconductors^11^,^25^ and the only one concerning PEDOT-based materials.

## Conclusion

High boiling point additives were used as a tool for tuning the structural, electrical and thermoelectric properties of PEDOT:Tos thin films. It was observed that processing PEDOT:Tos with such additives can result in more crystalline films, leading to a threefold maximum increase in conductivity, with respect to the pristine film. The Seebeck coefficient remained unaffected and the power factor therefore increases with the electrical conductivity. In order to examine the origin of the improvement, XPS measurements proved that carrier density is not affected, implying that conductivity should be driven solely by mobility changes. Indeed, in-plane mobility measurements confirmed the tight relation with conductivity. Lastly, we demonstrated that the behavior of mobility is dictated by the structural properties of the PEDOT:Tos films, and specifically by the thin film crystallinity combined to the preferential edge-on orientation of the PEDOT crystallites. This study underlines the substantial importance of structural fine-tuning through processing methods. Enhanced charge transport and improved thermoelectric performance are proven by an increase of the power factor from 25 to 78.5 μW/mK^2^ and can be readily obtained for PEDOT:Tos thin films following this methodology.

## Methods

### Materials and Processing

PEDOT:Tos films were synthesized *via in-situ* polymerization of 3,4-ethylene dioxythiophene (EDOT) monomers in the presence of iron(III) *p*-toluenesulfonate (Fe(Tos)_3_). The oxidant solution (40% in 1-butanol) was purchased from Heraus (Clevios B40), while EDOT and all other additives and solvents were purchased from Sigma-Aldrich and used without further purification. The EDOT monomers were added to the Fe(Tos)_3_ oxidant solution with an oxidant-to-monomer ratio equal to 2.3:1 based on the fact that 2 moles of oxidant are needed to polymerize the monomer, plus 0.3 moles to dope the system. The solution was stirred under ambient conditions for 12 hours. The oxidant/EDOT dispersion was spin-coated on 15 × 15 mm glass substrates at 1.5 krpm for 30 s to form 180 nm thick films. Films were thermally annealed at 100 °C for 15 min to initiate the polymerization reaction. Afterwards the films were sequentially washed with 1-butanol and ethanol, to remove the remaining oxidant, and dried under nitrogen flow. Films were further dried under vacuum overnight. In case of additive-containing films, the various solvent additives were introduced in the oxidant solutions before EDOT addition, at a constant volume fraction of 5% v/v with respect to the solution volume. The list of additives used in this study is presented in Table 1.

### Characterizations

Sheet resistance, *R*_□_, was measured directly on the film surface *via* a 4-point probe set-up utilizing a Lucas Lab S-302-4 station having *R* recorded with a Keithley 2450. The film thickness, *t*, measured with a Dektak XT stylus profilometer allowed calculation of the electrical conductivity *via σ* = 1/(*R*_□_*.t*).

The Seebeck coefficient was measured in a thin film geometry with a homemade setup^32^ utilizing two tungsten-pins to measure the voltage, *V*, (Keithley 2450), whilst the temperature, *T*, was recorded with 2 T-type thermocouples (Omega T-08). Substrates were coated with four 100 nm Au electrodes to ensure proper electrical contact. Temperature gradients were directly controlled through Peltier plates below the samples fixed using thermal pastes. Applied gradients were between 0–5 °C at room temperature and the resulting *V/T* slope was calculated as the Seebeck coefficient. The validity of the Seebeck coefficient determination was assured by using an optimal geometrical configuration for Seebeck coefficient measurements of thin film devices^33^ as detailed in Supporting Information (Figure S7).

Electrolyte-gated transistors were fabricated following the methodology proposed by Wei *et al*. and used for the calculation of the in-plane mobility of the PEDOT:Tos thin films^14^. A detailed description of the fabrication procedure and data analysis is provided in Supporting Information (see Figures S1 and S2).

The internal structure of the PEDOT:Tos thin films was probed using Grazing Incidence Wide Angle X-ray Scattering (GIWAXS). GIWAXS measurements were performed on the Dutch-Belgian Beamline (DUBBLE CRG), station BM26B, at the European Synchrotron Radiation Facility (ESRF), Grenoble, France^34^. The energy of the X-rays was 12 keV, the sample-to-detector distance and the angle of incidence, *α*_*i*_, were set at 11 cm and 0.16°, respectively. The diffracted intensity was recorded by a Frelon CCD camera and was normalized by the incident photon flux and the acquisition time (30 s). Flat field, polarization, solid angle and efficiency corrections were subsequently applied to the 2D GIWAXS images^35^. No modification of the scattering patterns were observed for 5 consecutive acquisitions allowing us to conclude that the samples are stable to radiation damage during measurements. The scattering vector *q* was defined with respect to the center of the incident beam and has a magnitude of *q* = (4π/*λ*)sin(*θ*), where 2*θ* is the scattering angle and *λ* is the wavelength of the X-ray beam. Herein we opted to present the wedge-shaped corrected images where *q*_*r*_ and *q*_*z*_ are the in-plane and near out-of-plane scattering vectors, respectively. The scattering vectors are defined as follows: *q*_*x*_ = (2π/*λ*)(cos(2*θ*_*f*_)cos(*α*_*f*_)-cos(*α*_*i*_)), *q*_*y*_ = (2π/*λ*)(sin(2*θ*_*f*_)cos(*α*_*f*_)), *q*_*z*_ = (2π/*λ*)(sin(*α*_*f*_) + sin(*α*_*i*_)), *q*_*r*_^2^ = *q*_*x*_^2^ + *q*_*y*_^2^, where *α*_*f*_ is the exit angle in the vertical direction and 2*θ*_*f*_ is the in-plane scattering angle, in agreement with standard GIWAXS notation^36^.

## Additional Information

**How to cite this article**: Petsagkourakis, I. *et al*. Structurally-driven Enhancement of Thermoelectric Properties within Poly(3,4-ethylenedioxythiophene) thin Films. *Sci. Rep.* **6**, 30501; doi: 10.1038/srep30501 (2016).

## Supplementary Material

Supplementary Information

## Figures and Tables

**Table 1:** Solvent additives and their respective boiling points, *T*_*b*_.

Additive	Tb [°C]	σ [S/cm]	S [μV/K]	μ [cm^2^/Vs]
No additive (Pristine)	—	230 ± 10	33 ± 5	0.92 ± 0.05
Acetonitrile (ACN)	82	275 ± 10	32 ± 5	—
Toluene (Tol)	110	300 ± 10	33 ± 5	1.00 ± 0.05
Chlorobenzene (CB)	137	310 ± 10	35 ± 5	1.18 ± 0.05
Dimethylformamide (DMF)	153	640 ± 10	35 ± 5	2.20 ± 0.05
Dimethylsulfoxide (DMSO)	189	605 ± 15	32 ± 5	2.17 ± 0.05
Ethylene Glycol (EG)	197	355 ± 10	32 ± 5	1.38 ± 0.05
Propylene Carbonate (PC)	242	165 ± 10	35 ± 5	—

The electrical conductivity, *σ*, the Seebeck coefficient, *S*, and the in-plane moblility, *μ*, of the corresponding PEDOT:Tos films are also reported.
