# Supplementary material for: Structurally-driven Enhancement of Thermoelectric Properties within Poly(3,4-ethylenedioxythiophene) thin Films
Source: Sci Rep. 2016 Jul 29;6:30501. doi: 10.1038/srep30501 (PMC4965772; doi:10.1038/srep30501)
Supplement: Supplementary Information [file srep30501-s1.pdf]

SUPPORTING INFORMATION

Structurally-driven Enhancement of Thermoelectric Properties within Poly(3,4-ethylenedioxythiophene) thin Films

Ioannis Petsagkourakis[1], Eleni Pavlopoulou[1,2], Giuseppe Portale[3], Bryan A. Kuropatwa[4], Stefan Dilhaire[4], Guillaume Fleury[1,]* & Georges Hadziioannou[1,]*

1. **In-plane mobility measurements:**

The in-plane mobility was determined by fabricating electrolyte gated transistors[1]. On top of PEDOT:Tos thin films deposited on glass substrates, gold electrodes of 100 nm were evaporated *via* thermal evaporation for the transistor source-drain and the gate electrodes. Afterwards, an ion gel solution in acetone comprised of poly(vinylidene fluoride-co-hexafluoropropylene) (P(VDF-HFP)) and 1-ethyl-3-methylimidazolium bis(trifluoromethylsulfonyl)amide ([EMI][TFSA]) ionic liquid was spin-coated at 1.5 krpm for 30 s and then annealed for 1hr at 50°C to remove the excess solvent. The ion gel film had a 6 μm thickness. From the output characteristics in **Figure S1**, we can clearly observe the field effects in the transistor. Conductance characteristics for various gate voltages are plotted in **Figure S2**. Mobility was calculated following the relationship $\mu = \frac{dg_d}{dV_G} \cdot \frac{L}{W \cdot C}$ using the slope of the linear region, a capacitance, *C*, of 12 μF/cm^2, a length, *L*, of 100 μm and a width, *W*, of 3mm.

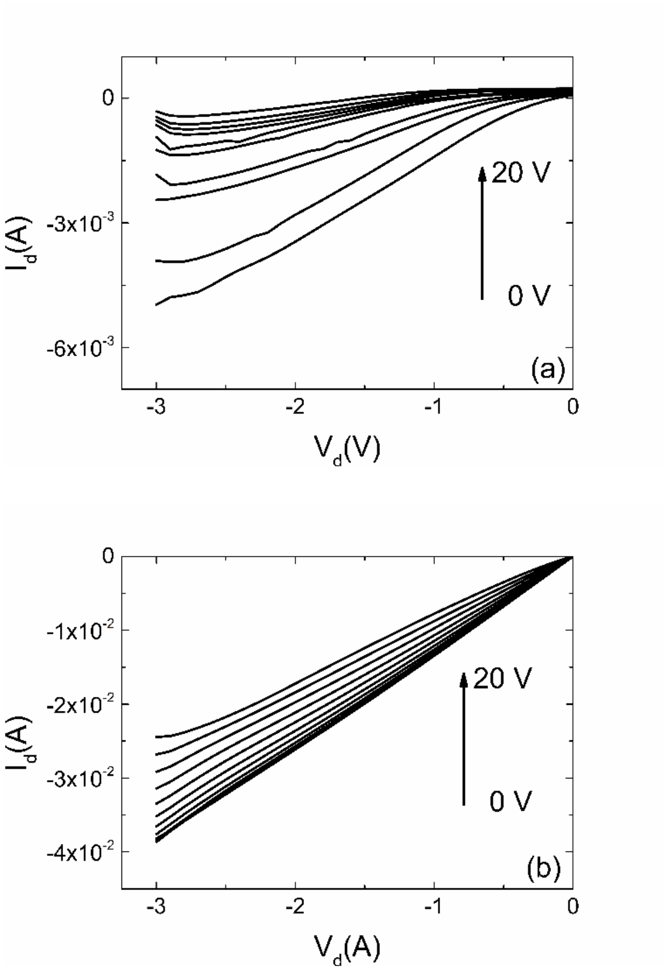

Figure S1. Output characteristics of the transistors. (a) for the pristine and (b) DMF added PEDOT:Tos films.

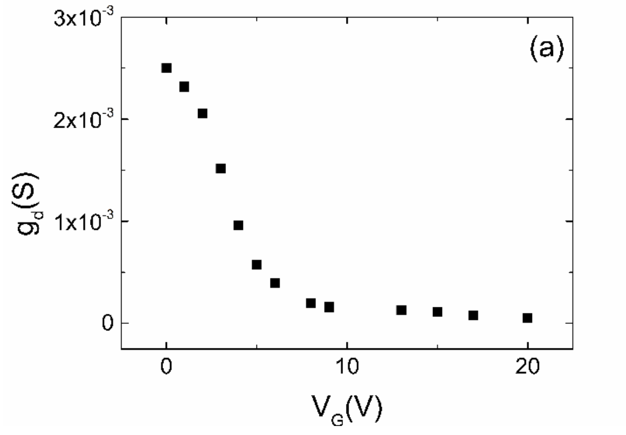

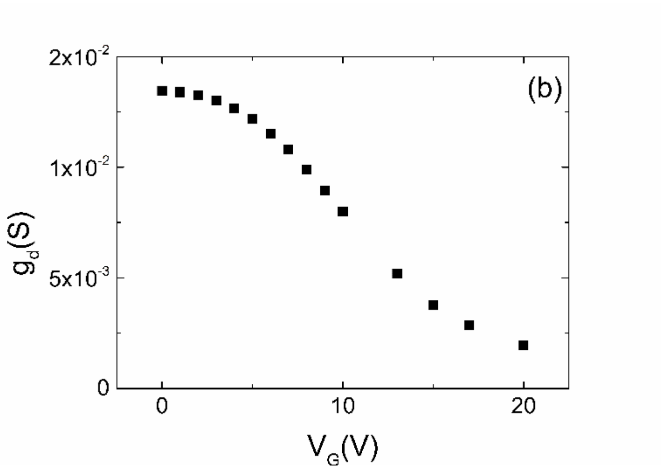

Figure S2. Conductance versus the gate voltage. (a) for the pristine and (b) DMF added PEDOT:Tos films.

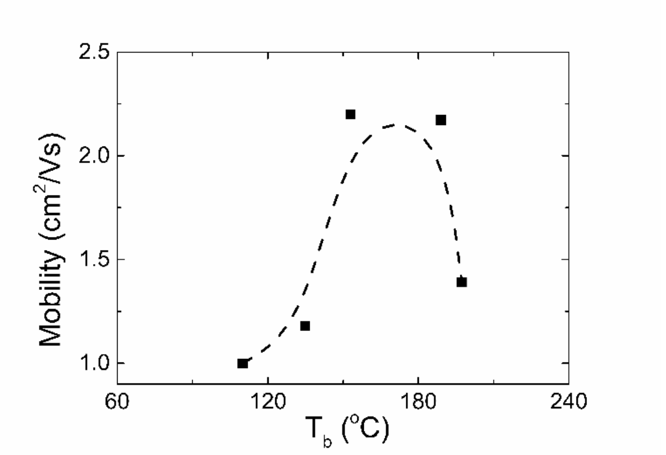

Figure S3. In-plane mobility of the PEDOT:Tos films plotted with respect to the boiling point of the various processed additives.

The calculated in-plane mobility is plotted against the boiling points of the various processed additives used in this study. In **Figure S3** one can observe that μ follows a bell-shaped trend, similar to that of σ versus T_b, as presented in **Figure 1**.

2. Structural characterization:

In **Figure S4** is the schematic for the unit cell of the PEDOT system examined herein. This Unit cell was proposed from Aamundstveit et al.[2]. The *b*-axis is along the substrate. The unit cell dimensions are a = 14 Å, b = 6.8 Å, c = 7.8 Å.

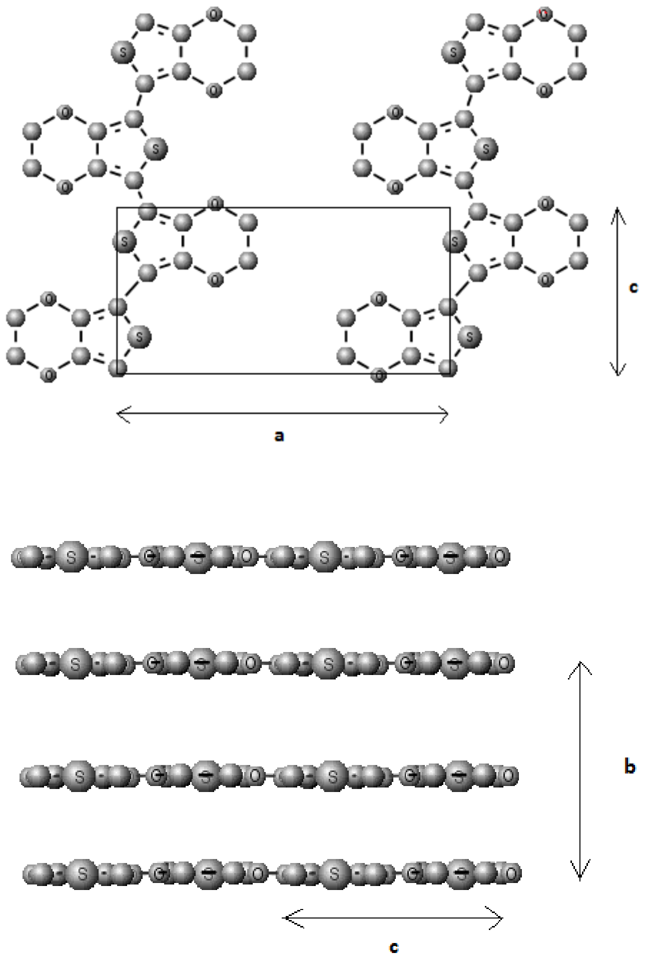

Figure S4. PEDOT:Tos structure with its unit cell.

Figure S5 displays the $I(\chi)\times sin(\chi)$ *vs* χ plots for the (100) peak. The area of the $I(\chi)\bullet sin(\chi)$ *vs* χ plots for the (100) peak was subsequently used as a measure of the relative crystallinity considering the PEDOT films as "in-plane powders" (i.e. isotropic in-plane orientation of the crystallites). Additionally an estimation of the edge-on fraction is possible through this plot. Given that $I(\chi)\times sin(\chi)$ plot represents the orientation distribution function of the (100) crystallites and considering that edge-on crystallites are oriented with a χ between 0°

and 45°, the fraction of edge-on oriented crystallites can be estimated from the ratio between the *I(χ)×sin(χ)* vs *χ* integral from 0° to 45° and the one from 0° to 90°.

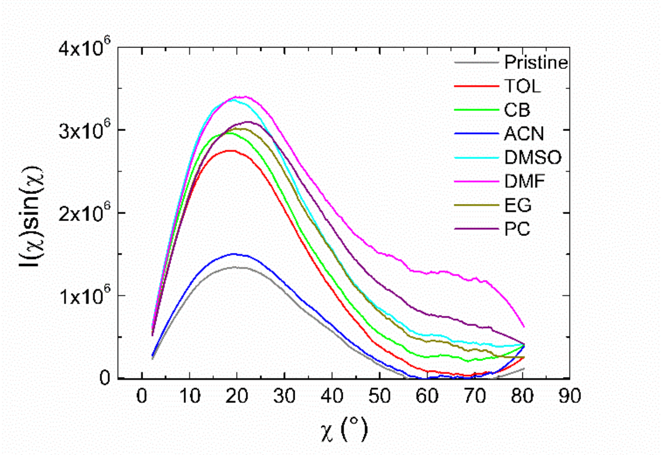

Figure S5. *I(χ)×sin(χ) vs χ* plots for the (100) peak of the various PEDOT:Tos thin films.

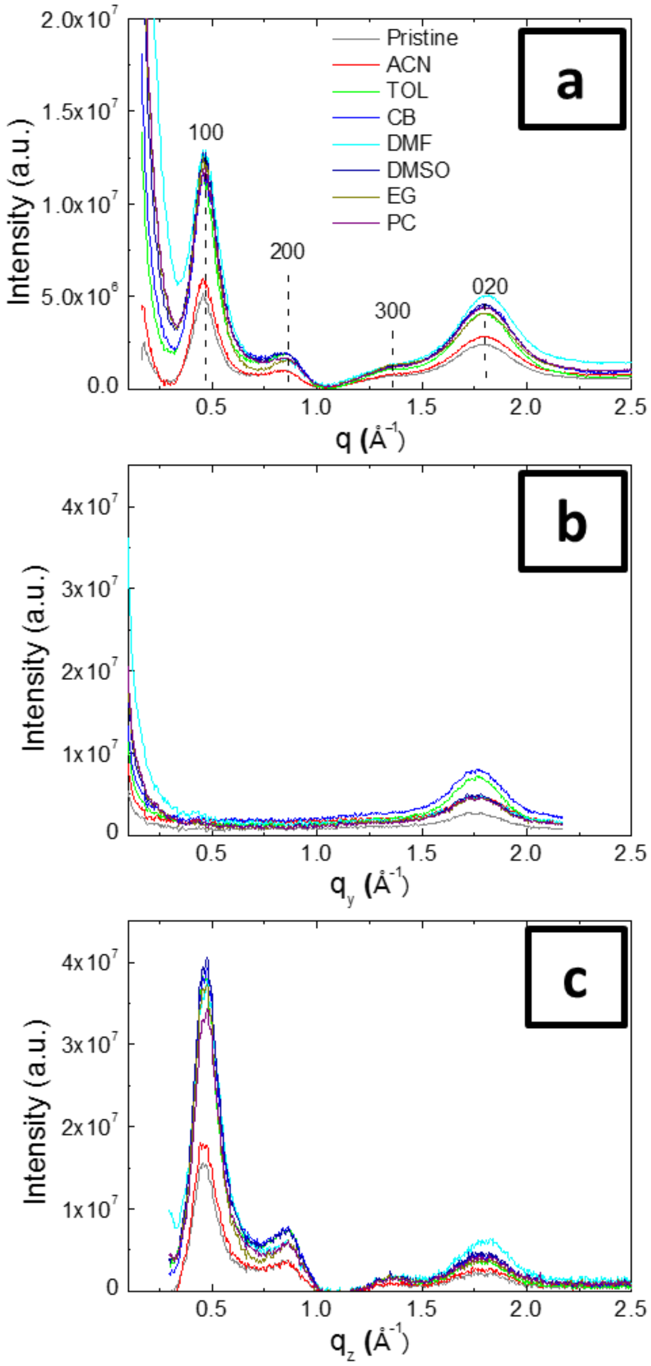

Figure S6. GIWAXS characterization of PEDOT:Tos thin films with additives. The 1D radially averaged intensity plots with respect to the scattering vector *q* (a) and the two intensity line cuts in the in-plane (q_y) (b) and across the near out-of-plane (q_z) (c) directions for PEDOT:Tos films synthesized with the various additives. In all cases the scattering intensity was normalized by the film thickness. All the intensity plots had the contribution of the substrate subtracted.

3. Seebeck coefficient measurements:

To minimize the potential errors on the Seebeck coefficient determination, the measurement configuration was optimized following a methodology described by van Reenen *et al.*[3]. Consequently the geometry of the Au electrodes and the channel length (gap between the Au electrodes) were chosen such as the channel and electrode aspect ratios (defined as the ratio of the electrode width to the channel and electrode length, respectively) are lower than 1. This geometrical configuration allow to minimize errors on the Seebeck coefficient determination[3]. To further asset the validity of the Seebeck coefficient determination, Ni foil was used for calibration of the Seebeck measurement set-up. In **Figure S7** the thermovoltage *vs.* temperature gradient for Ni foil calibrant is displayed. Value of the Seebeck coefficient obtained for this material was 19.5 ± 0.2 μV/K which is in accordance with reported values in the literature[4].

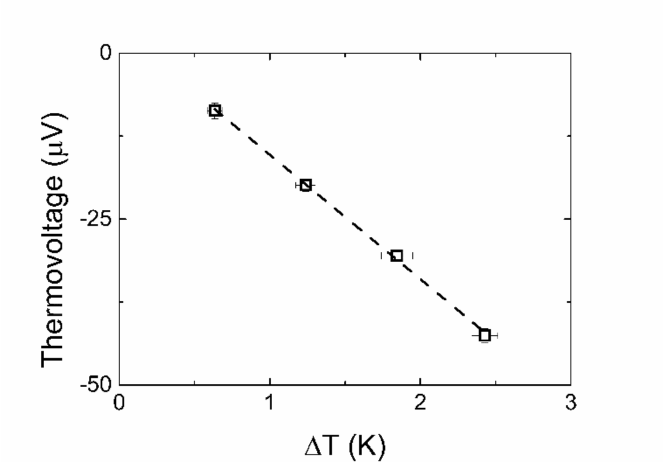

Figure S7. Thermovoltage versus temperature gradient for Ni foil calibrant. The dashed line is the linear fit, whose slope is used for the Seebeck coefficient calculation.

References

1. Wei, Q., Mukaida, M., Naitoh, Y. & Ishida, T. Morphological change and mobility

enhancement in PEDOT:PSS by adding co-solvents. *Adv. Mater.* **25,** 2831–2836 (2013).

2. Aasmundtveit, K. E. *et al.* Structure of thin films of poly(3,4-ethylenedioxythiophene). *Synth. Met.* **101,** 561–564 (1999).

3. Van Reenen, S., Kemerink, M. Correcting for contact geometry in Seebeck coefficient measurements of thin film devices. *Org. Electron.* **15**, 2250–2255 (2014).

4. Burkov, A. T. *Encyclopedia of Materials: Science and Technology*. *Encyclopedia of Materials: Science and Technology* (Elsevier, 2001).
